# The association of metabolic syndrome with telomere length as a marker of cellular aging: a systematic review and meta-analysis

**DOI:** 10.3389/fgene.2024.1390198

**Published:** 2024-07-09

**Authors:** Sulieman Ibraheem Shelash Al-Hawary, Abdullah Ali Alzahrani, Hatem Ghaleb Maabreh, Mohammed Abed Jawad, Salim B. Alsaadi, Noura Kareem Jabber, Ahmed Alawadi, Ali Alsalamy, Farideh Alizadeh

**Affiliations:** ^1^ Department of Business Administration, Business School, Al al-Bayt University, Mafraq, Jordan; ^2^ Department of Surgery, Taif University, Taif, Saudi Arabia; ^3^ Department of Dermatovenerology, Foreign Languages, Patrice Lumumba Peoples’ Friendship University of Russia (RUDN University), Moscow, Russia; ^4^ Department of Pharmaceutics, Al-Nisour University College, Baghdad, Iraq; ^5^ Department of Pharmaceutics, Al-Hadi University College, Baghdad, Iraq; ^6^ College of Health and Medical Technology, Al-Ayen University, Nasiriyah, Iraq; ^7^ College of Technical Engineering, The Islamic University, Najaf, Iraq; ^8^ College of Technical Engineering, The Islamic University of Al Diwaniyah, Al Diwaniyah, Iraq; ^9^ College of Technical Engineering, The Islamic University of Babylon, Babylon, Iraq; ^10^ College of Technical Engineering, Imam Ja’afar Al‐Sadiq University, Samawah, Iraq; ^11^ Department of Medicine, Tehran University of Medical Sciences, Tehran, Iran

**Keywords:** metabolic syndrome, telomere length, telomerase, aging, meta-analysis

## Abstract

**Background:**

It has been suggested that metabolic syndrome (MetS) accelerates the aging process, potentially contributing to the development of age-related complications. Available studies examining the relation of MetS to telomere length (TL), a putative biological marker of aging, have yielded inconclusive findings. This meta-analysis was performed to investigate the association between MetS and TL.

**Methods:**

A comprehensive systematic search was conducted in PubMed and Scopus databases to identify relevant literature published up to February 2024. Standard mean difference (SMD) and standardized beta coefficient (β) with their 95% confidence intervals (CI) were used as effect sizes to measure the associations using the random effects model.

**Results:**

A total of nine studies, comprising a total sample size of 8,606 participants, were eligible for the meta-analysis. No significant difference in mean TL was found between patients with and without MetS (SMD = −0.03, 95%CI = −0.17 to 0.10), with a significant heterogeneity across the studies (*I* ^2^ = 89.7.0%, *p* ≤ 0.001). In contrast, it was revealed that MetS is negatively related to TL (β = −0.08, 95%CI = −0.15 to −0.004). In the subgroup analysis, this finding was supported by the International Diabetes Federation (IDF) definition of MetS.

**Conclusion:**

This meta-analysis highlighted that MetS may be linked to a shorter TL. Additional studies are required to confirm this finding.

## Introduction

Metabolic syndrome (MetS) comprises a collection of interconnected components, including central obesity, hypertension, hyperglycemia, and dyslipidemia characterized by low high-density lipoprotein (HDL) cholesterol and elevated triglycerides (Alizadeh et al., 2018; Rashidbeygi et al., 2019). These factors collectively heighten the risk of developing chronic complications such as type 2 diabetes, cardiovascular diseases (CVD), and mortality (Razi et al., 2017). The prevalence of MetS is on the rise in both Western and developing countries, with the estimated impact on approximately 20%–25% of the global population (do Vale Moreira et al., 2020). Advanced age stands out as a robust predictor for MetS onset (Vishram et al., 2014). On the other side, MetS has been postulated to exert detrimental effects on the aging process, potentially leading to the development of age-related complications (Shen et al., 2023).

Aging is a process influenced by genetics, and telomeres, which are repetitive DNA sequences located at the ends of chromosomes, play a crucial role in preserving genome integrity and regulating cellular responses to environmental stressors (Fernandes et al., 2021). A shorter TL has been identified to be involved in the pathogenesis of chronic diseases (Weischer et al., 2013; Schneider et al., 2022). In normal cells, telomere length (TL) naturally reduces with each cell division (Whittemore et al., 2019). As a result, TL gradually shortens as age advances, making it a potential biological indicator of aging (Mather et al., 2011). Accelerated TL shortening can also be influenced by heightened exposure to chronic low-grade inflammation and oxidative stress, which are recognized as significant contributors to biological aging (Jurk et al., 2014; Gavia-García et al., 2021). Recent epidemiological studies have revealed significant relationships between multiple components of MetS and short TL, and elevated oxidative stress and inflammation have been proposed as potential mediators (Révész et al., 2015; Cheng et al., 2017). However, most previous studies have focused on individual components of MetS, and the evidence regarding the relationship between MetS as a whole and TL remains inconclusive. In studies by Dragović et al. (2021) and Devrajani et al. (2023), patients with MetS had a shorter TL, compared to individuals without MetS, while other studies failed to find an association between MetS and TL (Satoh et al., 2008; Uziel et al., 2013). The inconsistent results of previous studies may by derived from the differences in sample size, definition of MetS, and the age and sex of participants. Accordingly, this meta-analysis aimed to synthesize the available evidence to obtain a more comprehensive and conclusive understanding of the association between MetS and TL.

## Materials and methods

The present meta-analysis was carried out in accordance with the PRISMA guidelines (Moher et al., 2009).

### Search strategy

A thorough literature search was performed in PubMed and Scopus to identify relevant literature published until February 2024. The following keywords and related Medical Subject Headings (MeSH) were utilized in the search process: (telomere OR telomeres) AND (“metabolic syndrome” OR “insulin resistance syndrome” OR “syndrome x”). No language or date restriction was considered for the search. In addition, a manual search in the list of the references of the eligible studies was conducted to ensure that no studies were missed during the research process. The identified publications were imported into the Endnote software. Following the removal of any duplicate entries, the titles and abstracts of the articles were independently reviewed by two researchers. Any discrepancies or disagreements were addressed and resolved through a collaborative group discussion. Subsequently, the full-text articles were obtained, and the relevant information from each article was entered into an extraction sheet.

### Inclusion criteria

The inclusion criteria for the meta-analysis were as follows: 1) cross-sectional, cohort, and case-control studies that reported the relation of MetS (exposure) to TL (outcome) in adults, 2) the studies needed to report means and standard deviations (SD) of TL in patients with and without MetS or provided sufficient data to obtain standardized regression coefficients (β) along with their corresponding 95% confidence intervals (CI) for the association between MetS and TL. The analysis excluded studies focusing solely on individual components of MetS, as well as review articles, letters, comments, editorials, animal studies, and studies with unextractable data.

### Data extraction and quality assessment

The following data were extracted from the included studies: Region of study, the author’s name, outcomes examined, publication year, study design, method used for measuring TL, the criteria used for the definition of MetS, ethnicity, sex ratio (males %) and the mean age of participants, total sample size and the number of cases with MetS, the covariates controlled for in the analyses, as well as the means and SDs of TL in patients with and without MetS, along with the β and its corresponding 95%CI for the relationship between MetS and TL. To assess the methodological quality of the studies, we employed the Newcastle-Ottawa scale (NOS) (Wells et al., 2016). The NOS consists of three categories and eight items, with a star rating ranging from 0 to 9. The three categories, namely, comparison, selection, and result, are assigned 2 stars, 4 stars, and 3 stars, respectively. A score of 6 or higher indicates high quality, a score between 3 and 5 indicates medium quality, and a score below 3 indicates low quality. Data extraction and quality assessment were conducted independently by two authors for all studies. In case of any disagreements, a group discussion was held to resolve them.

### Statistical analysis

The difference in the TL between patients with and without MetS was evaluated using the standard mean difference (SMD) and 95%CI. Moreover, β and 95%CI were pooled to examine the association between MetS and TL. The *I* ^2^ statistic was applied to evaluate the level of statistical heterogeneity among the analyzed publications; *I* ^2^ ≥ 50% or *p*-value <0.05 were considered as statistically significant heterogeneity (Hosseinzadeh et al., 2020; Emami et al., 2021). Since there was a significant heterogeneity, the analysis were performed using the DerSimonian and Laird (DL) random effects model (DerSimonian and Kacker, 2007). Subgroup analysis by the definition of MetS, ethnicity of participants, and adjustment for covariates (yes vs. no) was done to find possible sources of the observed heterogeneity. Since TL length measured using qPCR methods vastly differs other methods, only studies that used qPCR methods to measure TL were examined for the subgroup analysis. Meta-regression analyses were conducted to evaluate whether the pooled results are affected by sample size, quality of studies, mean age, and sex ratio (male %) of participants. Publication bias was tested using the Egger’s regression test (Peters et al., 2006). Statistical tests were performed using STATA software, version 14.0, developed by Stata Corporation in College Station, TX, United States.

## Results

### Study characteristics

**FIGURE 1:**
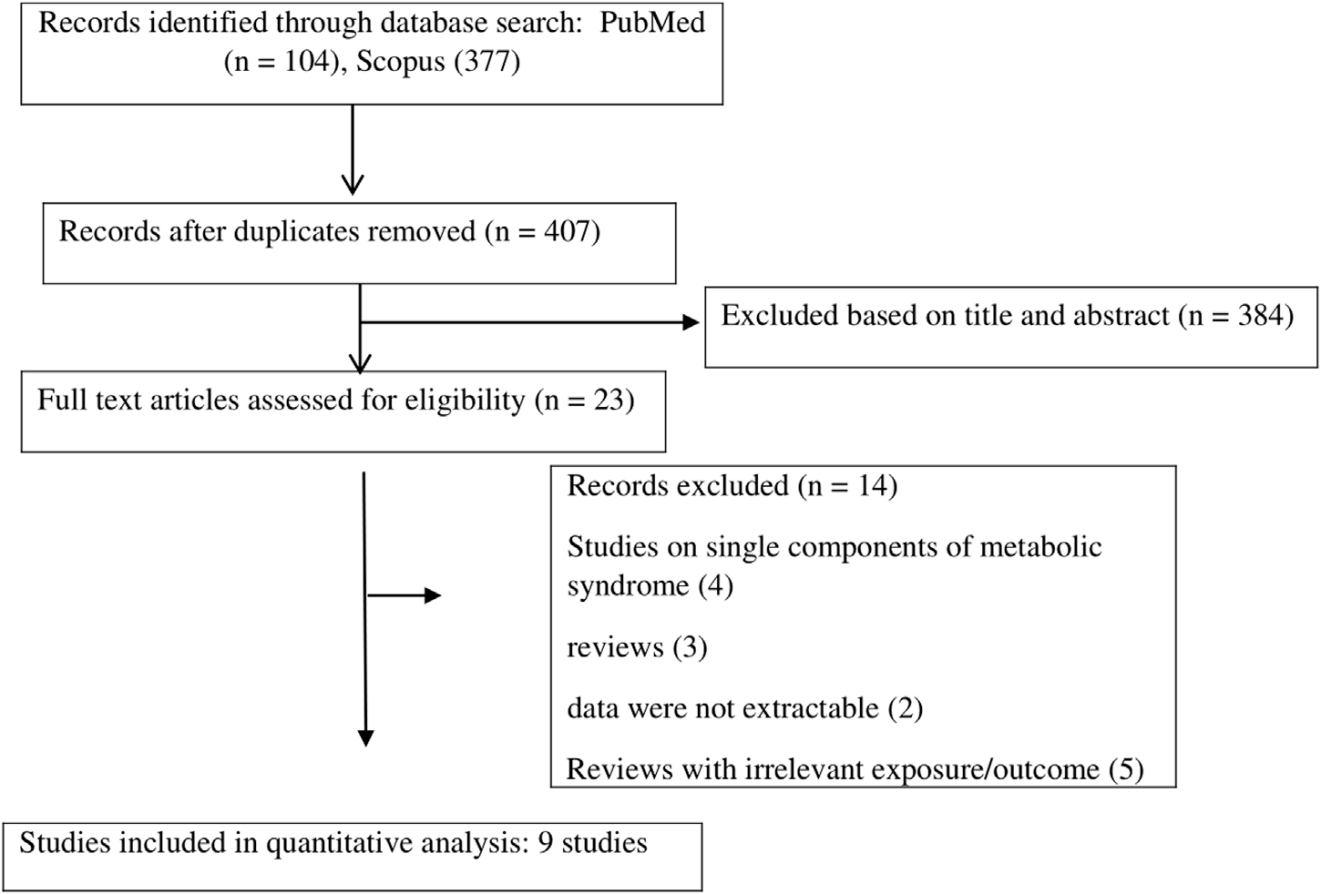
Flow chart for studies selection.

The systematic literature search identified 481 publications, out of which 74 were identified as duplicates. After evaluating the titles and abstracts, 384 articles were excluded. This process resulted in 23 potentially relevant studies that underwent full-text evaluation. During the full-text screening, 14 additional studies were excluded for various reasons, such as being review studies, focusing on single components of MetS, having unextractable data, or having irrelevant exposure/outcome. Finally, a total of nine publications (Satoh et al., 2008; Uziel et al., 2013; Rehkopf et al., 2016; Iglesias Molli et al., 2017; Dragović et al., 2021; Lejawa et al., 2021; Peng et al., 2021; Huang et al., 2022; Devrajani et al., 2023) spanning from 2008 to 2023, and involving a combined sample size of 8,606 participants, were incorporated into the meta-analysis. The screening process of studies is depicted in Figure 1. All studies employed a cross-sectional design. There were five studies on Caucasians (Uziel et al., 2013; Rehkopf et al., 2016; Iglesias Molli et al., 2017; Dragović et al., 2021; Lejawa et al., 2021) and 4 studies on Asians (Satoh et al., 2008; Peng et al., 2021; Huang et al., 2022; Devrajani et al., 2023). The mean age of participants ranged from 31.82 ± 3.68 to 64.1 ± 11.4 years. Four studies used Adult Treatment Panel III (ATP III) (Satoh et al., 2008; Rehkopf et al., 2016; Iglesias Molli et al., 2017; Devrajani et al., 2023), 3 studies used a guideline developed by the International Diabetes Federation, American Heart Association, and the National Heart, Lung, and Blood Institute (IDF/AHA/NHLBI) (Uziel et al., 2013; Lejawa et al., 2021; Huang et al., 2022), one study used IDF (Dragović et al., 2021), and one study applied the Chinese Guidelines on Prevention and Treatment of Dyslipidemia in Adults (Peng et al., 2021) for the definition of MetS. TL was assessed using the polymerase chain reaction (PCR) in seven studies (Uziel et al., 2013; Rehkopf et al., 2016; Iglesias Molli et al., 2017; Dragović et al., 2021; Lejawa et al., 2021; Huang et al., 2022; Devrajani et al., 2023), the Southern blotting method in one study (Peng et al., 2021), and the fluorescence *in situ* hybridization (Flow-FISH) method in another study (Satoh et al., 2008). Data for estimating the mean difference in TL between patients with and without MetS was presented in seven studies (Satoh et al., 2008; Uziel et al., 2013; Iglesias Molli et al., 2017; Dragović et al., 2021; Lejawa et al., 2021; Peng et al., 2021; Devrajani et al., 2023) and data for estimating the association of MetS with TL using the standardized beta regression (β) was available in eight studies (Satoh et al., 2008; Uziel et al., 2013; Rehkopf et al., 2016; Iglesias Molli et al., 2017; Dragović et al., 2021; Lejawa et al., 2021; Huang et al., 2022; Devrajani et al., 2023). Based on the NOS, the analyzed publications demonstrated a medium to high level of methodological quality, with scores ranging from 4 to 9 (Supplementary Table S1). The characteristics of the included publications are outlined in Table 1.

**TABLE 1:** Characteristics of studies.

References	Year	Study design	Location	No. of participants	MetS cases, n (%)	Age (rang or mean ± sd)	Definition of MetS	TL assessment	Effect sizes for outcomes	Adjustment
Dragovic	2021	Cross-sectional	Serbia	24	11 (45.8%)	50 ± 17.77	IDF	qPCR	Standardized β regression for the association of MetS with TL	Not adjusted
									Mean difference of relative TL between patients with and without MetS	
Uziel	2013	Cross-sectional	Israel	62	19 (30.2%)	56.8 ± 13.97	IDF/AHA/NHLBI	qPCR	Standardized β regression for the association of MetS with TL	Not adjusted
									Mean difference of relative TL between patients with and without MetS	
Huang	2021	Cross-sectional	Taiwan	121	33 (27/2%)	43.2 ± 11.5	IDF/AHA/NHLBI	qPCR	Standardized β regression for the association of MetS with TL	Adjusted for sex, education, body mass index, cigarette use, marriage
Lejawa	2021	Cross-sectional	Poland	48	22 (45.8%)	31.82 ± 3.68	IDF/AHA/NHLBI	qPCR	Standardized β regression for the association of MetS with TL	Adjusted for age, smoking and drinking
									Mean difference of relative TL between patients with and without MetS	
Peng	2021	Cross-sectional	China	344	184 (53%)	53.01 ± 13.54	Chinese Guidelines on Prevention and Treatment of Dyslipidemia in Adults	Southern blot	Mean difference of relative TL between patients with and without MetS	Adjusted for age
									Odds ratio for the association of TL with the odds of MetS in logistic regression model	
Rehkopf	2016	Cross-sectional	United States	7,252	NR	45.7 (20–84)	ATP III	qPCR	Standardized β regression for the association of MetS with TL	Adjusted for race/ethnicity, gender, foreign birthplace, education, class of work, income, marital status, age, smoking, physical activity, white blood cells, and proportions of white blood cells
Satoh	2008	Cross-sectional	Japan	57	31 (54.3%)	64.1 ± 11.4	ATP III	flow-FISH	Standardized β regression for the association of MetS with TL	Not adjusted
									Mean difference of relative TL between patients with and without MetS	
Molli	2017	Cross-sectional	Argentina	400	102 (25.5%)	46.76 ± 15.47	ATP III	qPCR	Standardized β regression for the association of MetS with TL	Adjusted for age, pack-years smoked and physical activity
									Mean difference of relative TL between patients with and without MetS	
Devrajani	2023	Cross-sectional	Pakistan	298	149 (50%)	54.8 ± 7.5	ATP III	qPCR	Standardized β regression for the association of MetS with TL	Not adjusted
									Mean difference of relative TL between patients with and without MetS	

NR, not reported; qPCR, quantitative polymerase chain reaction; Flow-FISH, fluorescence *in-situ* hybridization; MetS, metabolic syndrome; TL, telomere length.

### Quantitative analysis

**FIGURE 2:**
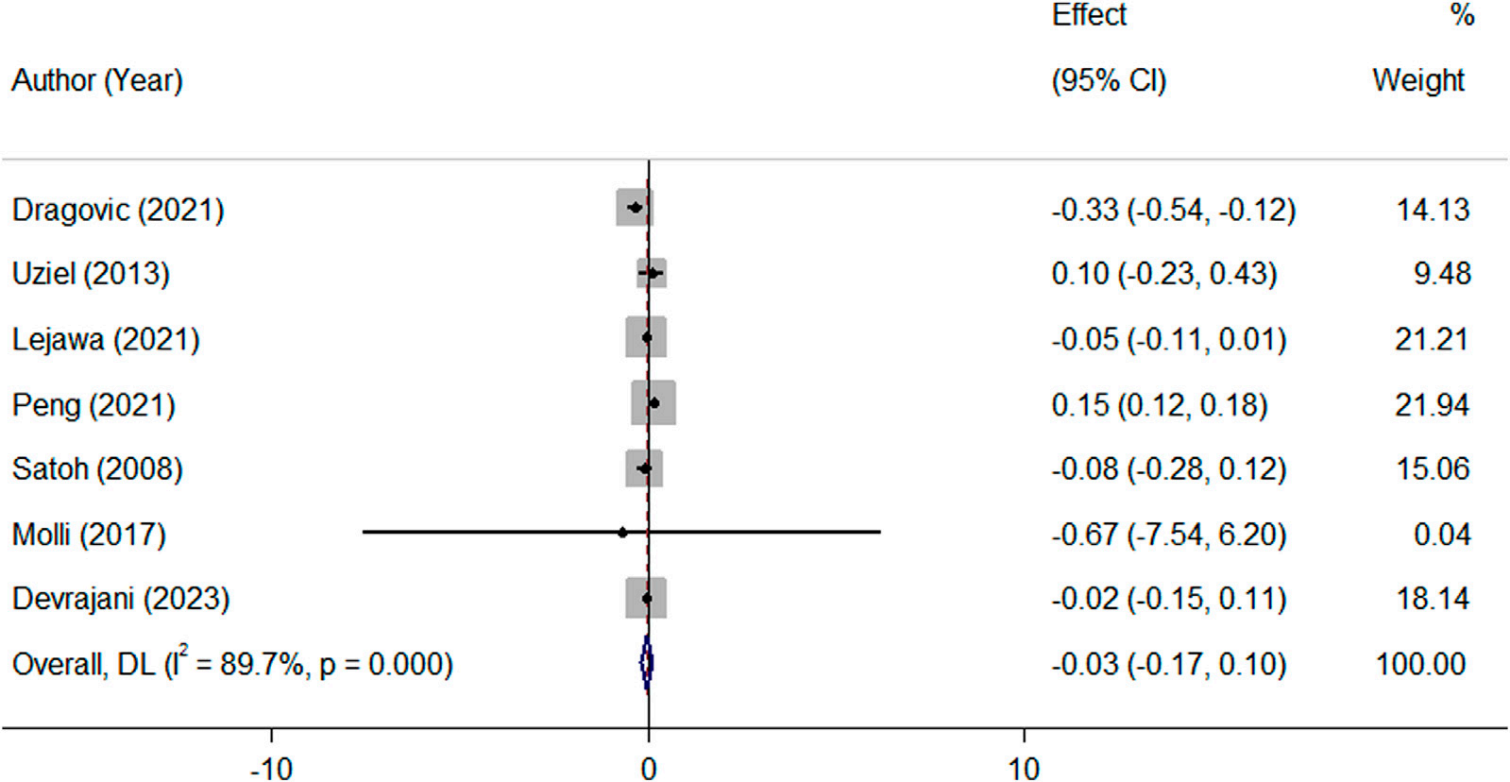
Overall meta-analysis of the standard mean difference (SMD) of telomere length in patients with MetS, compared to people without MetS.

**FIGURE 3:**
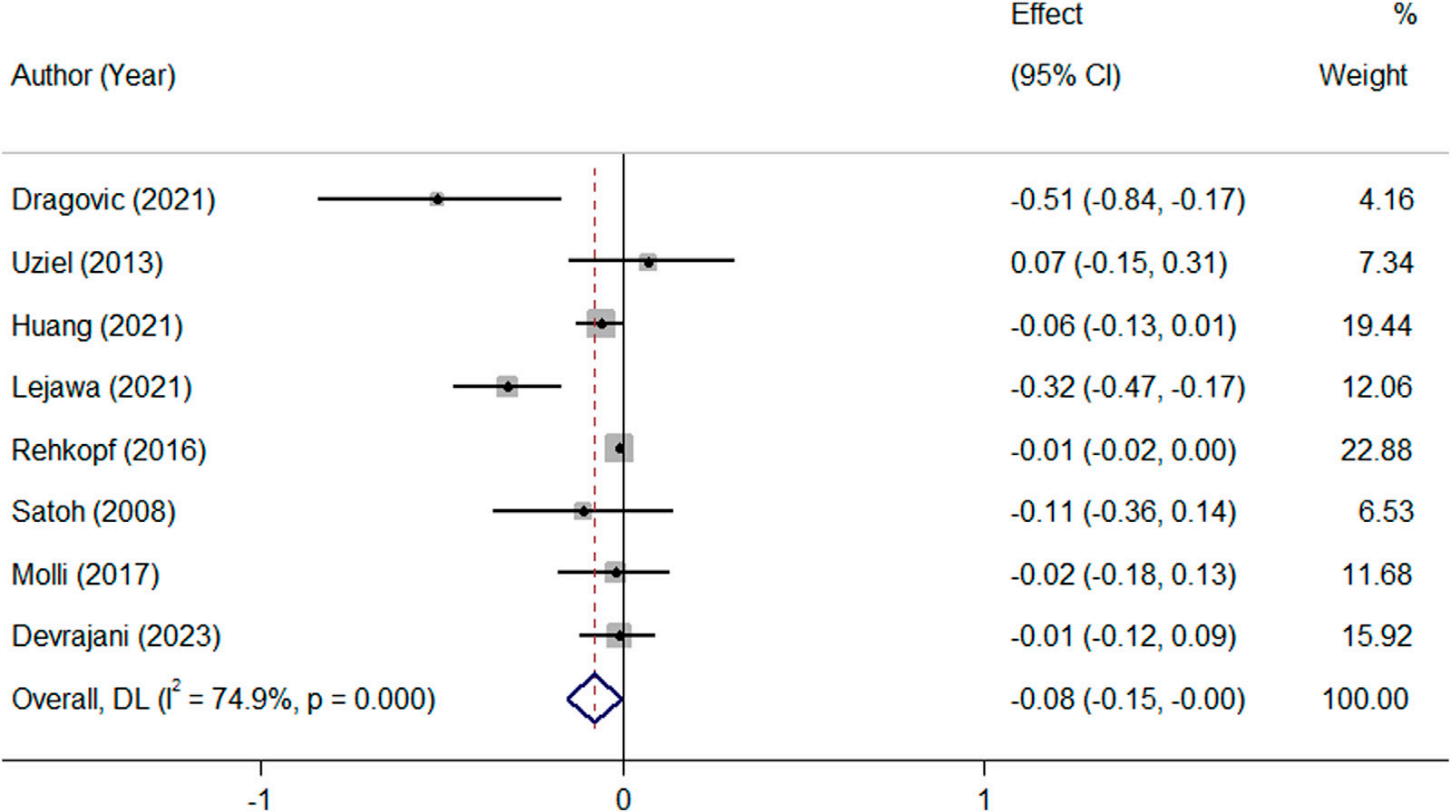
Overall meta-analysis of the standardized beta regression coefficients (β) for the association between MetS and telomere length.

Pooled analysis of all available studies identified no significant difference in mean TL between patients with and without MetS (SMD = −0.03, 95%CI = −0.17 to 0.10; Figure 2), with a notable presence of heterogeneity among the studies (*I* ^2^ = 89.7.0%, *p* ≤ 0.001). In the subgroup analysis, this finding was not affected by ethnicity and the level of adjustment for covariates (Table 2). It was revealed that MetS is negatively related to TL (β = −0.08, 95%CI = −0.15 to −0.004; Figure 3), with a notable presence of heterogeneity among the studies (*I* ^2^ = 89.7.0%, *p* ≤ 0.001). In the subgroup analysis, this finding was supported by the IDF definition of MetS, while was not significant in subgroup analysis by ethnicity and the level of adjustment for covariates (Table 2).

**TABLE 2:** Overall and subgroup analysis for the association between telomere length and metabolic syndrome in studies that used qPCR to measure telomere length.

Effect size for the association				Test of association		Test of heterogeneity	
Standardized β regression for the association of MetS with TL (outcome)	Criteria used to select studies for subgroup analyses	Subgroups (references)	Studies	β	95%CI	I^2^ (%)	*p*
	Ethnicity	Caucasian (Uziel et al., 2013; Rehkopf et al., 2016; Iglesias Molli et al., 2017; Dragović et al., 2021; Lejawa et al., 2021)	5	−0.13	−0.29 to 0.03	84.3	<0.001
		Asian (Huang et al., 2022; Devrajani et al., 2023)	2	−0.05	−0.10 to 0.01	0.0	0.43
	Definition of MetS	IDF (Dragović et al., 2021)	1	−0.51	−0.85 to −0.17	-	-
		IDF/AHA/NHLBI (Uziel et al., 2013; Lejawa et al., 2021; Huang et al., 2022)	3	−0.11	−0.31 to 0.08	82.8	0.003
		ATP III (Rehkopf et al., 2016; Iglesias Molli et al., 2017; Devrajani et al., 2023)	3	−0.01	−0.02 to 0.003	0.0	0.99
	Adjustment for covariates	Yes (Rehkopf et al., 2016; Iglesias Molli et al., 2017; Lejawa et al., 2021; Huang et al., 2022)	4	−0.08	−0.18 to 0.01	83.7	<0.001
		No (Uziel et al., 2013; Dragović et al., 2021; Devrajani et al., 2023)	3	−0.11	−0.37 to 0.15	77.1	0.01

Mean difference of relative TL between patients with and without MetS	Criteria used to select studies for subgroup analyses	Subgroups (references)		SMD	95%CI	I^2^ (%)	*p*
	Ethnicity	Caucasian (Uziel et al., 2013; Iglesias Molli et al., 2017; Dragović et al., 2021; Lejawa et al., 2021)	4	−0.11	−0.30 to 0.08	57.6	0.07
		Asian (Devrajani et al., 2023)	1	−0.02	−0.15 to 0.11	-	-
	Definition of MetS	IDF (Dragović et al., 2021)	1	−0.33	−0.54 to −0.12	-	-
		IDF/AHA/NHLBI (Uziel et al., 2013; Lejawa et al., 2021)	2	−0.05	−0.10 to 0.01	80.3	<0.001
		ATP III (Iglesias Molli et al., 2017; Devrajani et al., 2023)	2	−0.02	−0.15 to 0.11	0.0	0.85
	Adjustment for covariates	Yes (Iglesias Molli et al., 2017; Lejawa et al., 2021)	2	−0.05	−0.11 to 0.01	0.0	0.86
		No (Uziel et al., 2013; Dragović et al., 2021; Devrajani et al., 2023)	3	−0.10	−0.33 to 0.14	71.9	0.02

### Meta-regression analysis and publication bias

**FIGURE 4:**
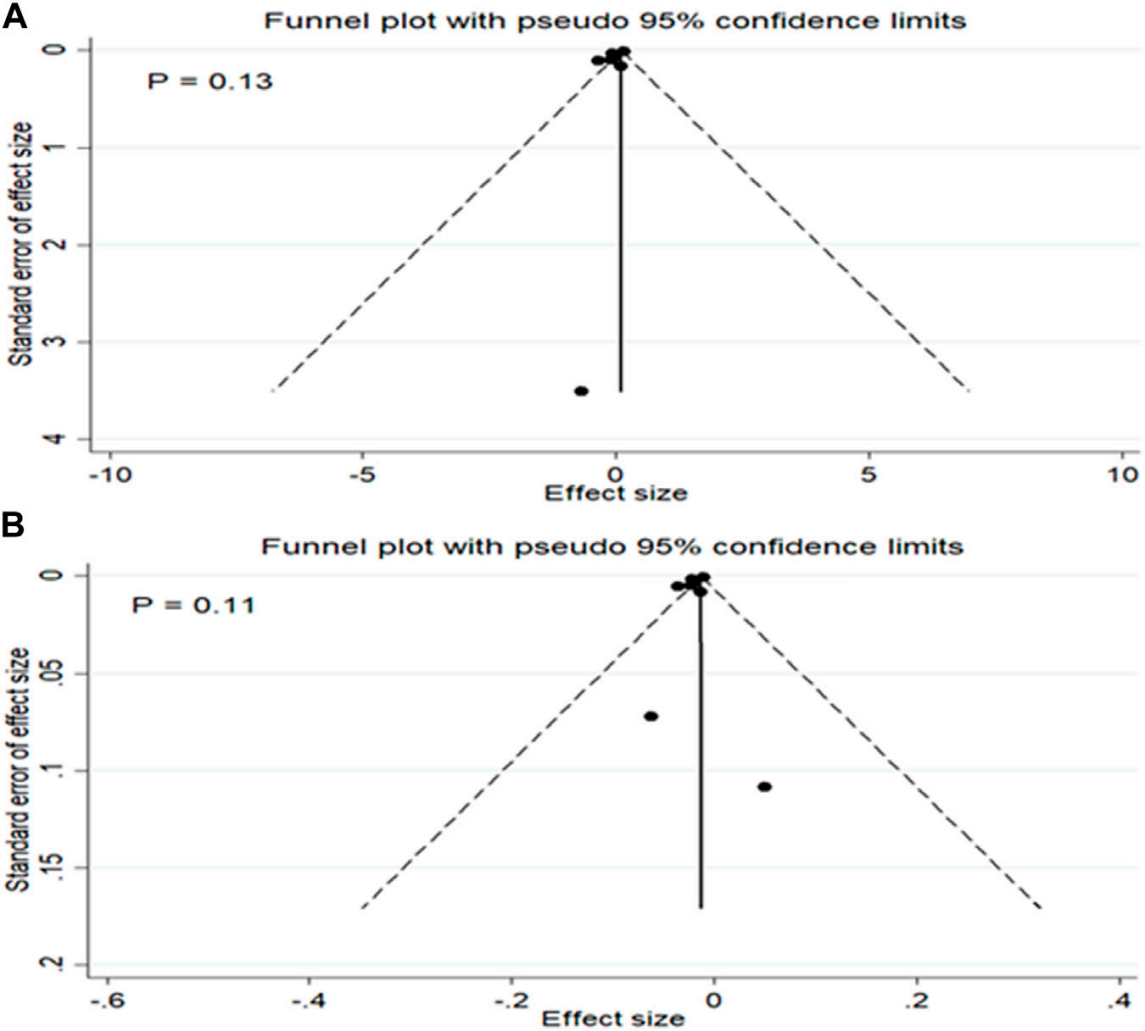
Publication bias for the mean difference in TL between patients with and without MetS **(A)** and for the association between MetS and TL **(B)**.

No substantial evidence of publication bias was found for any of the analyses, as determined by the Egger’s regression test (Figure 4). In the meta-regression analyses, the associations were not affected by sample size of studies, quality of studies, and the age and sex ratio (% males) of participants (all *p*-values ≥0.10).

## Discussion

The objective of this meta-analysis was to investigate the association between MetS and TL. The findings revealed that MetS is negatively linked to TL. This association was not affected by the sample size, as well as age and gender distribution among participants.

Available studies investigating the association between MetS and TL have yielded inconclusive results. A cross-sectional study in 2023 found that uncontrolled MetS appeared to have worsening effects on TL (Devrajani et al., 2023). Another study investigated the longitudinal associations between MetS components and telomere shortening revealed that TL was inversely associated with the number of MetS components, suggesting that MetS may accelerate telomere shortening (Révész et al., 2015). In contrast, in the cross-sectional study conducted by Uziel et al., no significant correlation was found between the average length of telomeres and the presence of MetS or its individual components in liver transplant recipients (Uziel et al., 2013). The present meta-analysis identified that the heterogeneity in the results of previous studies could be due to the differences in the definition of metabolic syndrome, the level of adjustment for covariates, and ethnicity of the studied populations. This analysis suggested a significant negative association between MetS and TL, indicating that MetS may contribute to accelerated cellular aging. Furthermore, some studies have also indicated a bidirectional relationship, where shorter telomeres may predispose individuals to the development of MetS and its traits (Révész et al., 2014). These findings suggest a complex interplay between MetS and TL, with potential implications for understanding the pathophysiology of MetS and its related health outcomes. Understanding the interplay between MetS and TL may have implications for the development of preventive and therapeutic personalized strategies targeting age-related diseases associated with MetS.

It is notable that while there was no significant mean difference in TL between individuals with and without MetS, a significant inverse relationship was observed between MetS and TL using pooled β regression coefficients. In studies that investigated the mean difference in TL between patients with and without MetS, the net mean differences in TL between patients with and without MetS were presented without adjustment for potential covariates, whereas several studies that used regression analysis to examine the association of MetS with TL adjusted the results for potential covariates. Accordingly, differences in adjustment for covariates have resulted in two contradictory results for pooled mean differences and B coefficients, emphasizing that future studies examining the relationship between MetS and TL should consider confounding factors to provide a more comprehensive understanding of this association.

Mechanistically, the inverse association between MetS and TL could be mediated by detrimental impacts of oxidative stress, chronic inflammation, insulin resistance, obesity, and ayslipidemia on telomere biology, including shelterin complex, a protein complex that protects telomeres from degradation and maintains telomere length, and telomerase activity, the enzyme responsible for maintenance of the length of telomere (Huzen et al., 2014; Kirchner et al., 2017; Gavia-García et al., 2021; Loh et al., 2023). Reduced telomerase activity has often been observed in individuals with MetS or its components (Devrajani et al., 2023). MetS is often characterized by increased oxidative stress, which refers to an imbalance between the production of reactive oxygen species (ROS) and the body’s antioxidant defenses (Spahis et al., 2017). Oxidative stress can lead to DNA damage, including telomere shortening by inhibiting telomerase activity (Von, 2000). Telomeres have a high sensitivity to oxidative stress-mediated damage. Elevated levels of oxidative stress in individuals with MetS may accelerate telomere attrition (Gavia-García et al., 2021). MetS is closely associated with chronic low-grade inflammation. Inflammatory processes can promote telomere shortening through the release of pro-inflammatory cytokines and other mediators (Wong et al., 2014). The sustained inflammatory response observed in MetS could contribute to the accelerated loss of TL (Sutherland et al., 2004). Insulin resistance, a hallmark of MetS, can affect telomere length through various mechanisms. Insulin resistance leads to hyperinsulinemia and increased insulin-like growth factor 1 (IGF-1) levels, which can activate signaling pathways associated with cellular proliferation and aging, potentially affecting telomere dynamics (Kolb et al., 2023). MetS often involves dyslipidemia, which may contribute to telomere shortening by promoting oxidative stress, inflammation, and endothelial dysfunction, all of which impact telomere integrity (Liu and Li, 2015). Lower HDL in patients with MetS increases oxidation and inflammation (Holven et al., 2013). It is important to note that these mechanisms are interconnected and can influence each other. Furthermore, while these mechanisms are supported by scientific evidence, the exact relationships and underlying processes are still an active area of research.

Obesity, a hallmark of MetS, has been associated with reduced telomerase activity, the enzyme responsible for maintaining and elongating telomeres. Reduced telomerase activity can result in accelerated telomere shortening over time (Shin, 2019). In obesity, there is an imbalance in adipokine production, with increased production of pro-inflammatory adipokines such as leptin and decreased production of anti-inflammatory adipokines such as adiponectin (Vendrell et al., 2004). This imbalance can contribute to inflammation and oxidative stress, both of which can impact telomere length (Zhu et al., 2011). Obesity, independent of its comorbidities, reduces TL by inhibiting the telomerase activity and up-regulating the TRF1, a negative regulator of the shelterin complex, thereby altering the function of telomeres (Grun et al., 2018). Furthermore, Mendelian randomization studies have supported that higher BMI, independent of other MetS components, has a negative association with TL, equivalent to 1.70 years of age-related TL change (Loh et al., 2023). Obesity, is known to increase systemic inflammation and oxidative stress, which can lead to shorter TL (Mundstock et al., 2015). It is established that engaging in physical activities has a beneficial impact on both mental and physical wellbeing (Yagmaee, 2021). Exercise can mitigate some of the TL reductions in patients with obesity, by reducing chronic inflammation and oxidative stress (Arsenis et al., 2017; Irandoust and Taheri, 2018). Physical activity has been associated with longer telomeres in various studies (Ludlow et al., 2013; Arsenis et al., 2017). Exercise-induced changes in body composition, oxidative stress, inflammation, and metabolic profiles in obese individuals can positively influence telomere integrity by reducing chronic inflammation and oxidative stress (Denham et al., 2016; Shin, 2019). Weight loss is also linked to telomere lengthening, especially in subjects with shortest telomeres at baseline (Carulli et al., 2016). Moreover, a adherence to a healthy diet rich in antioxidants from fruits, vegetables, whole grains and healthy fats, while limiting processed meats and alcohol, may help preserve TL and may promote healthy aging in the context of MetS (Paul, 2011). Currently, there are no studies directly examining the impact of physical activity on TL in individuals with MetS. We suggest that future studies investigate the influence of exercise interventions on TL in MetS patients. Longitudinal studies exploring the impacts of continuous physical activity and weight control on TL in people with MetS could offer crucial causal insights that are presently absent in the field.

Therefore, clinicians can encourage patients to combine regular physical activity with a healthy diet to protect TL shortening and improve overall health outcomes related to short TL. Public health policymakers can also promote physical activity as a preventive measure for accelerated biological aging in MetS patients. There is solid evidence for policy effectiveness in some areas such as school-based and infrastructural policies (Gelius et al., 2020). The world health organization (WHO) report (Organization, 2023) advocates for investment in promoting healthy lifestyles in the older population to encourage active healthy ageing and increase healthy life expectancy. Based on the WHO report encouraging employers to implement workplace wellness programs that incorporate physical activity, such as exercise breaks, fitness classes, or on-site gyms, promoting flexible work schedules to allow employees to be active during the day, implementing school-based programs that provide physical education and opportunities for physical activity, increasing access to sports facilities and infrastructure that encourages active transportation and outdoor activities, and launching public awareness campaigns to highlight the benefits of physical activity for healthy aging could be effective to improve physical activity (Organization, 2023).

To our current understanding, this study is the first meta-analysis to examine the correlation between MetS and TL. The study exhibits several strengths, including a relatively large sample size, the absence of publication bias, and the use of subgroup and meta-regression analyses to examine the associations. However, it is important to recognize certain limitations of the present meta-analysis. First, it is important to consider substantial heterogeneity among the included studies. The subgroup analysis indicated that the observed heterogeneity could be attributed to variations in the level of adjustment for covariates, ethnic background, and the definition of MetS. However, differences in sample size, age, and gender distribution among participants did not contribute to the heterogeneity based on the meta-regression analysis. Moreover, the heterogeneity of the studies included in the meta-analysis is a significant limitation. Some studies were based on participants with specific disorders and treatments, such as HIV/AIDS, liver transplantation, coronary artery disease, type 2 diabetes, and major depressive disorders. These conditions can independently influence TL, which may affect the association between TL and MetS. This heterogeneity limits the generalizability of the findings and highlights the need for future studies to control for these confounding factors. Second, some studies included effect sizes based on raw estimates without accounting for potential covariates, which can introduce bias and affect the overall finding. Lastly, it should be noted that due to the observational nature of the studies included in this meta-analysis, it is not possible to establish causal relationships based on the findings. There are several potential scenarios to consider: telomere shortening may interfere with metabolic function, metabolic health could impact telomere length, or the association between the two factors may be mutually influential in a bidirectional manner. Future studies with a prospective design can provide more robust evidence regarding the relation of MetS to TL, allowing for a better understanding of the directionality of the association. Moreover, performing interventional studies to investigate the effects of interventions targeting either TL or MetS could elucidate if changes in one factor have an impact on the other.

## Conclusion

In conclusion, this meta-analysis presented evidence supporting the inverse relationship between MetS and TL. These findings suggest that MetS may have a detrimental impact on TL, potentially contributing to accelerated cellular aging and increased risk of age-related complications. Additional research, specifically utilizing a prospective cohort design, is needed to gain a better understanding of the association between MetS and TL. Future studies are recommended to further elucidate the underlying biological mechanisms and explore potential interventions to mitigate the negative effects of MetS on TL.

## Data Availability

The original contributions presented in the study are included in the article/Supplementary Material, further inquiries can be directed to the corresponding author.
