# Supplementary material for: The association of metabolic syndrome with telomere length as a marker of cellular aging: a systematic review and meta-analysis
Source: Front Genet. 2024 Jul 9;15:1390198. doi: 10.3389/fgene.2024.1390198 (PMC11263212; doi:10.3389/fgene.2024.1390198)
Supplement: Supplementary file 1 [file Table1.DOCX]

**Supplemental Table 1.** Quality assessment of studies included in this systematic review and meta-analysis

|  | Representativeness of the sample | Sample size | Non-respondents | Ascertainment of exposure | Study controls for age | Study controls for any additional factor | Ascertainment of outcome | Statistical test | **Total score** |
| --- | --- | --- | --- | --- | --- | --- | --- | --- | --- |
| Dragovic, 2021 | NA | NA | * | * | NA | NA | ** | NA | 4 |
| Uziel, 2013 | NA | NA | * | * | NA | NA | ** | NA | 4 |
| Huang, 2021 | NA | * | * | * | NA | * | ** | * | 7 |
| Lejawa, 2021 | * | NA | * | * | * | * | ** | * | 8 |
| Peng, 2021 | NA | * | * | * | * | NA | ** | * | 7 |
| Rehkopf, 2016 | * | * | * | * | * | * | ** | * | 9 |
| Satoh, 2008 | NA | NA | * | * | NA | NA | ** | NA | 4 |
| Molli, 2017 | * | * | * | * | * | * | ** | * | 9 |
| Devrajani, 2023 | * | * | * | * | NA | NA | ** | NA | 6 |

NA: Not Applicable

**Online Supporting Material**

**Supplemental Table 2.** Subgroup analysis based on fixed effects models for the association between DASH diet and the risk of chronic kidney disease.

|  | |  | | | | | | Effect sizes (n) | I^2^ (%) | P _within_  _heterogeneity_ | ES (95%CI) | P _between_  _heterogeneity_ |
| --- | --- | --- | --- | --- | --- | --- | --- | --- | --- | --- | --- | --- |
|  | | | | | | | | |  |  |  |  |
|  | Overall | |  | | | | | 10 | 69.9 | 0.001 | 0.76 (0.64 -0.91) |  |
|  | Study design | | | | | | |  |  |  |  | <0.001 |
|  |  | | Prospective | | | | | 7 | 70.6 | 0.002 | 0.77 (0.63 -0.94) |  |
|  |  | | Cross-sectional | | | | | 3 | 77.0 | 0.01 | 0.72 (0.38 -1.36) |  |
|  | Geographical region | | | | | | |  |  |  |  | <0.001 |
|  |  | | USA | | | | | 5 | 71.8 | 0.007 | 0.93 (0.70 -1.22) |  |
|  |  | | Iran  Korea | | | | | 4  1 | 0  - | 0.48  - | 0.60 (0.50 -0.73)  0.78 (0.65-0.94) |  |
|  | DASH assessment | | |  | |  | | |  |  |  | <0.001 |
|  |  | | FFQ | | | | | 6 | 69.7 | 0.006 | 0.73 (0.59 -0.89) |  |
|  |  | | 24 h recall | | | | | 4 | 77.6 | 0.004 | 0.86 (0.50 -1.46) |  |
|  | Energy as covariate | | | | | | |  |  |  |  | <0.001 |
|  |  | | Used | | | | | 7 | 72.9 | 0.001 | 0.65 (0.50- 0.85) |  |
|  |  | | Not-used | | | | | 3 | 67.6 | 0.04 | 0.96 (0.73 -1.26) |  |
|  | Adjustment for BMI | | | |  | | |  |  |  |  | <0.001 |
|  |  | | Adjusted effect size | | | | | 7 | 64.4 | 0.01 | 0.75 (0.64-0.88) |  |
|  |  | | Non-adjusted effect size | | | | | 3 | 83.8 | 0.002 | 0.88 (0.34-2.26) |  |
|  |  | |  | | | | |  |  |  |  |  |
|  | Adjustment for smoking | | | | | |  |  |  |  |  | <0.001 |
|  |  | | Adjusted effect size | | | | | 9 | 69.0 | 0.001 | 0.73 (0.62-0.87) |  |
|  |  | | Non-adjusted effect size | | | | | 1 | - | - | 1.48 (0.84-2.61) |  |
|  |  | |  | | | | |  |  |  |  |  |
|  |  | |  | | | | |  |  |  |  |  |

**
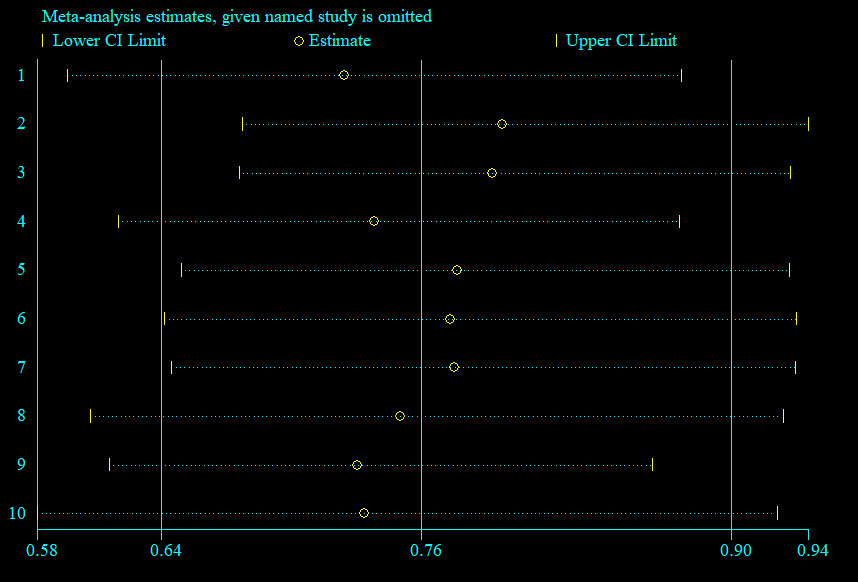
**

**Online Supporting Material**

**Supplemental Figure 4.** Sensitivity analysis of the association between DASH diet and the risk of chronic kidney disease
